# Mental health and chest CT scores mediate the relationship between COVID-19 vaccination status and seroconversion time: A cross-sectional observational study in B.1.617.2 (Delta) infection patients

**DOI:** 10.3389/fpubh.2022.974848

**Published:** 2022-10-19

**Authors:** Wen Zhang, Qian Chen, Jinghong Dai, Jiaming Lu, Jie Li, Yongxiang Yi, Linqing Fu, Xin Li, Jiani Liu, Jinlong Liufu, Cong Long, Bing Zhang

**Affiliations:** ^1^Department of Radiology, The Affiliated Drum Tower Hospital of Nanjing University Medical School, Nanjing, China; ^2^Medical Imaging Center, Affiliated Drum Tower Hospital, Medical School of Nanjing University, Nanjing, China; ^3^Institute of Medical Imaging and Artificial Intelligence, Nanjing University, Nanjing, China; ^4^Department of Pulmonary and Critical Care Medicine, The Affiliated Drum Tower Hospital of Nanjing University Medical School, Nanjing, China; ^5^Department of Infectious Disease, The Affiliated Drum Tower Hospital of Nanjing University Medical School, Nanjing, China; ^6^Department of Infectious Diseases, The Second Hospital of Nanjing, The Affiliated Hospital of Nanjing University of Chinese Medicine, Nanjing, China; ^7^Institute of Brain Science, Nanjing University, Nanjing, China

**Keywords:** COVID-19, SARS-CoV-2, B.1.617.2 Delta variant, vaccination, mental health, seroconversion time

## Abstract

**Background:**

The coronavirus disease (COVID-19) pandemic, which has been ongoing for more than 2 years, has become one of the largest public health issues. Vaccination against severe acute respiratory syndrome coronavirus 2 (SARS-CoV-2) infection is one of the most important interventions to mitigate the COVID-19 pandemic. Our objective is to investigate the relationship between vaccination status and time to seroconversion.

**Methods:**

We conducted a cross-sectional observational study during the SARS-CoV-2 B.1.617.2 outbreak in Jiangsu, China. Participants who infected with the B.1.617.2 variant were enrolled. Cognitive performance, quality of life, emotional state, chest computed tomography (CT) score and seroconversion time were evaluated for each participant. Statistical analyses were performed using one-way ANOVA, univariate and multivariate regression analyses, Pearson correlation, and mediation analysis.

**Results:**

A total of 91 patients were included in the analysis, of whom 37.3, 25.3, and 37.3% were unvaccinated, partially vaccinated, and fully vaccinated, respectively. Quality of life was impaired in 30.7% of patients, especially for mental component summary (MCS) score. Vaccination status, subjective cognitive decline, and depression were risk factors for quality-of-life impairment. The chest CT score mediated the relationship of vaccination status with the MCS score, and the MCS score mediated the relationship of the chest CT score with time to seroconversion.

**Conclusion:**

Full immunization course with an inactivated vaccine effectively lowered the chest CT score and improved quality of life in hospitalized patients. Vaccination status could influence time to seroconversion by affecting CT score and MCS score indirectly. Our study emphasizes the importance of continuous efforts in encouraging a full vaccination course.

## Introduction

The coronavirus disease (COVID-19) pandemic, which has been ongoing for more than 2 years, has become one of the largest public health issues and has affected the lives of most people worldwide (1). Quality of life domains and wellbeing outcomes have been worsened due to the pandemic in several parts of the world. Vaccination against severe acute respiratory syndrome coronavirus 2 (SARS-CoV-2) infection is one of the most important interventions to mitigate the COVID-19 pandemic (2). Available evidence has demonstrated that COVID-19 vaccines are effective in the prevention of severe complications of COVID-19 and death (2, 3), moreover, these vaccines are effective against infection with the different variants of COVID-19 (4). Clinical trials have demonstrated that vaccine efficacy can significantly affect disease outcomes, as vaccination can prevent symptomatic COVID-19 in 65.9–83% of people and severe illness or intensive care unit (ICU) admission in 90% to 100% of infected patients (5–7). A previous study reported that the 28-day seroconversion rate in subjects exceeded 80% (8). Additionally, the incidence of COVID-19 in the unvaccinated population was more than double (20.07%) that in the fully vaccinated population (9).

In China, people are generally vaccinated with inactivated vaccines (2). Accumulated evidence suggests that inactivated vaccines can efficiently, although not fully, protect against SARS-CoV-2 infection and, more importantly, prevent severe illness progression (10). Previous studies have confirmed the protective effect of the inactivated vaccine against the pathogenesis of SARS-CoV-2 mutant strains (2, 9), such as the Delta Variant (B.1.617.2).

COVID-19 infection directly affects not only overall survival but also central nervous system function, leading to acute neuropsychiatric symptoms (11). COVID-19 also indirectly affects the neuropsychiatric state (12). The contraction of the COVID-19 virus, spectrum of infection, environment and social situations, and social isolation also significantly affect the mental state of patients. COVID-19 patients have reported an increased sense of anxiety and reduced quality of life 6 months after hospital admission (13).

Whether vaccination status with an inactivated vaccine affects the seroconversion time in patients during hospital admission remains unclear. Thus, we performed a real-world study that analyzed patients' clinical, neuropsychiatric assessment, laboratory examination and chest CT image data in a designated hospital in Nanjing. Our study aimed to describe how the relationship between vaccination status and seroconversion time is affected by mental health and chest CT imaging findings during hospitalization and its effect on the pathogenesis of SARS-CoV-2 mutant strains.

## Methods

### Patients

This study was conducted between July 21, 2021, and September 15, 2021. Ninety-one COVID-19 patients with SARS-CoV-2 B.1.617.2 infection were enrolled at the Second Hospital of Nanjing, which is a specialized hospital for infectious diseases that was responsible for the admission of infected patients throughout Jiangsu Province during the SARS-CoV-2 B.1.617.2 variant pandemic. The inclusion criteria were as follows: (a) confirmed COVID-19 diagnosis via nasopharyngeal or oropharyngeal swab; (b) confirmation of the presence of the B.1.617.2 (Delta) variants by high-throughput whole-genome sequencing; (c) hospital admission and disease management; and (d) age ≥18 years. Patients were excluded from the study if they met any of the following exclusion criteria: (a) an unconfirmed etiology of pneumonia; (b) no available computed tomography (CT) scans; (c) an unconfirmed vaccination status (d) refusal to provide consent or (e) unable to complete questionnaires. The vaccination detail of 21 subjects was unknown. Fifteen subjects were excluded due to illiterate or unable to complete questionnaires. Finally, a total of 91 patients were enrolled in this study. The severity of disease was classified into three grades: mild, moderate, and severe, according to the Chinese management guideline for COVID-19 (14). None of the patients in the acute phase exhibited progression to severe disease, and all cases were mild or moderate. All patients received adequate medical support during hospitalization. This study was approved by the ethics committee of the Second Hospital of Nanjing, Nanjing, China (Nr: 2021-LS-ky031). Written informed consent was obtained from all participants.

### Clinical evaluation

The baseline day was defined as the day of the initial positive for new coronavirus nucleic acid by using the SARS-CoV-2 specific RT-PCR test. Nasal swabs were sampled at an interval of 24 h for examination of viral titer during hospitalization. The diagnostic criteria for viral seroconversion were two consecutive nasal swab samples negative by the SARS-CoV-2 RT-PCR test. Time to seroconversion was defined as the time from baseline day to viral seroconversion. All patients completed a structured electronic questionnaire independently within three days of hospital admission. The contents of the questionnaire included basic information (age, sex, education level, date of birth, height, weight, smoking status), medical history, and neuropsychological tests (cognition, quality of life, anxiety, depression and posttraumatic stress). The investigator was blinded to medical history during the data acquisition to avoid selection bias. Clinical symptoms, treatments, initial laboratory indicators, and chest CT imaging were extracted from the electronic medical system. Initial laboratory and imaging results were defined as the first-time examination within 24 h after admission.

### Definition of vaccination status

We categorized the patients into 3 groups, unvaccinated, partially vaccinated, and fully vaccinated, according to their immunization history (15). Patients were considered unvaccinated if they had never been vaccinated or had received 1 dose of the vaccine with a time interval between the first dose and illness onset of <14 days. Patients who had received 1 vaccine dose with an interval of more than 14 days or received 2 vaccine doses with an interval between the second dose and illness onset of <14 days were considered partially vaccinated. Patients were considered fully vaccinated if they received 2 doses of the vaccine and the time interval between the last vaccine dose and illness onset was more than 14 days.

### Cognition, quality of life, and emotional state evaluations

Cognitive impairment was evaluated both objectively and subjectively. Objective cognitive impairment was assessed by the Mini Mental Status Examination (MMSE), a widely used psychological test of global cognitive function among elderly individuals; it includes tests for orientation, attention, memory, language and visual-spatial skills (16). The maximum MMSE score is 30 points, and a score below 25 suggests cognitive impairment. Subjective cognitive decline (SCD) was assessed by a newly developed questionnaire consisting of nine items (17), that examine global memory functioning and the ability to perform daily activities. Higher scores indicate a higher risk of SCD.

Health-related quality of life was evaluated with the 36-item Short Form (SF-36), a well-validated 36-item questionnaire (18). The questionnaire includes one multi-item scale that assesses eight health concepts: physical function, role physical, bodily pain, general health, validity, social function, role-emotional and mental health. The total scores of the physical component summary (PCS) and mental component summary (MCS) range from 0 to 100 points each, with higher levels indicating a better health condition. Total scores below 117 are considered to indicate impairment according to norm-based scoring in elderly Chinese individuals.

Posttraumatic stress was assessed by the Posttraumatic Stress Disorder Checklist-5 (PCL-5) (19). The PCL-5 is a self-report instrument for the assessment of distress associated with the 20 symptoms of posttraumatic stress disorder (PTSD), with scores > 32 indicating clinically relevant PTSD. Depression and anxiety were evaluated with the Hospital Anxiety and Depression Scale (HADS) (20). This score consists of anxiety (HADS-A) and depression (HADS-D) subscales, each of which contains seven items scored from 0 to 3 points. The scores of each subscale range from 0 to 21, with higher scores indicating a larger number of intensive anxiety- and depression-related symptoms. Scores >7 indicate mild disorder, and scores >10 indicate clinically meaningful anxiety disorder or depression.

### CT protocol

Chest CT scans were performed using a 64-MDCT scanner (Brilliance 64, Philips Healthcare, Cleveland, Ohio, USA) or a 16-MDCT scanner (Aquilion TSX-101A, Toshiba, Tokyo, Japan). All chest CT scans were obtained with the patient in the supine position and scanning was performed at end-inspiration. The following parameters were used: tube voltage of 120 kVp, automatic tube current modulation (ATCM), reconstruction matrix of 512 × 512 pixels, section thickness of 1.5 mm, and interval of 1.5 mm.

### Evaluation of the CT score

The CT score, a widely used evaluation method by radiologists to quantify the extent of lung abnormalities, was determined for every CT scan (21, 22). Each lung lobe was scored from 0 to 5 based on the percentage of infection: score of 0, no involvement; score of 1, <5% involvement; score of 2, 6–25% involvement; score of 3, 26–49% involvement; score of 4, 50–75% involvement; and score of 5, >75% involvement. The CT score was derived from the sum of the individual lobar scores, resulting in CT scores ranging from 0 to 25 for each CT scan.

### Statistical analysis

**Figure 1:**
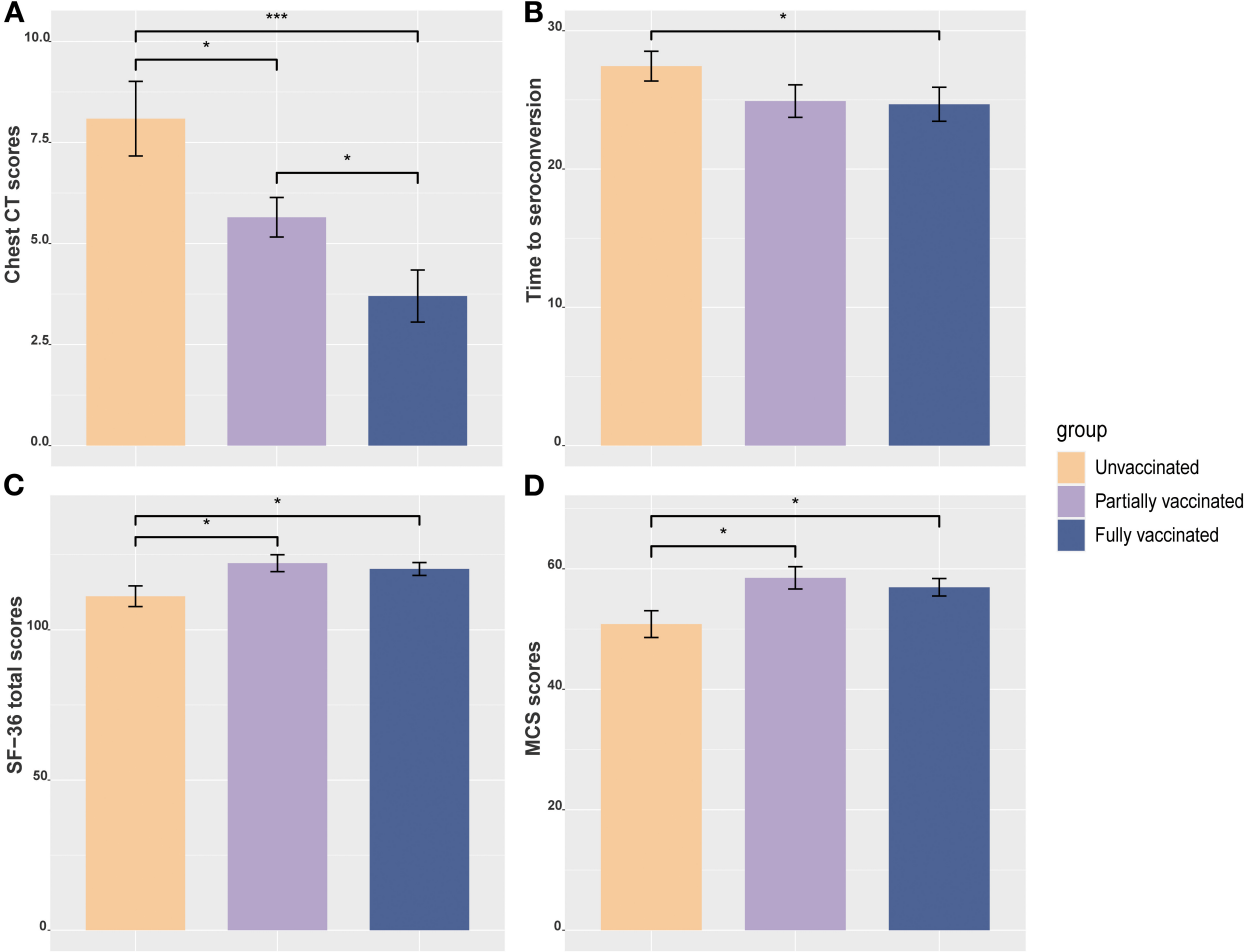
Bar plots among different vaccination status groups with the mean and standard error of the mean are shown. **(A)** Between-group differences in chest CT scores. **(B)** Between-group differences in time to seroconversion. **(C)** Between-group differences in SF-36 total score. **(D)** Between-group differences in MCS score. *P < 0.05; ***P < 0.001. MCS, mental component summary.

Statistical analyses were performed with IBM SPSS Statistics Software (version 26; IBM, New York, USA). Categorical variables are expressed as frequencies, and continuous variables are described as means ± standard deviations (SDs). The chi-square or Fisher's exact test was used to compare categorical variables. We used analyses of covariance (ANCOVA) to compare the group differences in laboratory findings, chest CT scores, and multiple psychological assessments. Risk factors related to impaired quality of life were analyzed by univariate regression. Variables that were significantly associated with the dependent variable in the univariate analyses (*P* < 0.05) were included in a multivariable regression model. Odds ratios (ORs) were reported with 95% confidence intervals (CIs). The SF-36 total score, PCS score, and MCS score were correlated with the CT score and time to seroconversion using partial correlation analyses. Partial correlation is a method used to describe the relationship between two variables whilst taking away the effects of another variable, or several other variables, on this relationship. To ensure our results are not affected by demographic features, age, sex and education levels were controlled as covariates in partial correlation analyses (23). After establishing that vaccination status has effects on the chest CT score, SF-36 scale scores and time to seroconversion (Figure 1), we applied mediation model analyses to test the hypothesis that the relationship between vaccination status and time to seroconversion are mediated by chest CT score or SF-36 scale scores. Mediation analyses are employed to understand a known relationship by exploring the underlying mechanism or process by which one variable influences another variable through a mediator variable (24). Mediation analysis was performed using the PROCESS SPSS macro toolbox. The indirect effect in the mediation model was estimated using 5,000 bias-corrected bootstraps with 95% CIs, and *P* < 0.05 indicated statistical significance.

## Results

### Characteristics of the patients

A total of 91 patients were included in the analysis, of whom 34 (37.3%), 23 (25.3%), and 34 (37.3%) were unvaccinated, partially vaccinated, and fully vaccinated, respectively. The inactivated vaccines used were the CoronaVac (Sinovac Biotech, Beijing, China), BBIBP-CorV (Sinopharm, Beijing, China), and KCONVAC (BioKangtai, Shenzhen, China) vaccines, accounting for 57.4, 33.8, and 8.8% of the vaccine doses, respectively. There was no significant difference in sex, education level, smoking status, alcohol consumption, medical history or major symptoms of COVID-19 (Table 1) among the groups. Fully vaccinated patients were younger and had higher diastolic blood pressure (DBP) than unvaccinated patients. Vaccinated patients had a higher lymphocyte level, shorter time to seroconversion, and lower chest CT score during the immediate hospitalization period after adjustment for age, sex and education than the other vaccination status groups (Table 2 and Figures 1A,B). There was no significant difference in the viral load between unvaccinated and fully vaccinated patients, represented by the PCR Ct value of the N gene (*P* = 0.297) or ORF1ab gene (*P* = 0.270).

**Table 1:** Characteristics and clinical features of COVID-19 patients with different vaccination statuses.

	**Unvaccinated** **(*n* = 34)**	**Partially** **vaccinated** **(*n* = 23)**	**Fully** **vaccinated** **(*n* = 34)**	***p*** **-value**
Age (years)	64.5 ± 7.9	56.1 ± 5.6	55.3 ± 5.2	<0.001^*^
Sex (male)	10 (29.4)	11 (47.8)	16 (47.1)	0.264
Education level (years)	7.0 ± 3.2	8.7 ± 3.7	8.8 ± 4.1	0.202
Current smoking	2 (5.9)	3 (13.0)	3 (8.8)	0.588
BMI (kg/m^2^)	23.9 ± 2.5	23.5 ± 3.3	25.0 ± 2.9	0.200
SBP (mmHg)	129.5 ± 15.9	129.2 ± 13.6	131.1 ± 18.6	0.889
DBP (mmHg)	80.6 ± 12.0	84.6 ± 8.5	87.3 ± 11.1	0.042^*^
**Medical history**				
Hypertension	16 (47.1)	6 (26.1)	10 (29.4)	0.194
Diabetes	8 (23.5)	2 (8.7)	7 (20.6)	0.346
Dyslipidemia	9 (26.5)	5 (21.7)	8 (23.5)	0.953
Coronary heart disease	4 (11.8)	1 (4.3)	1 (2.9)	0.494
COPD	1 (2.9)	1 (4.3)	0	–
Cerebrovascular disease	0	1 (4.3)	0	–
Chronic liver disease	0	1 (4.3)	1 (2.9)	–
Renal insufficiency	1 (2.9)	0	0	–
Malignant tumor	4 (11.8)	2 (8.7)	1 (2.9)	0.472
**Signs and symptoms**				
Fever	23 (67.6)	12 (52.2)	16 (47.1)	0.211
Cough	22 (64.7)	14 (60.9)	19 (55.9)	0.757
Fatigue	10 (29.4)	2 (8.7)	7 (20.6)	0.160
Stuffy or runny nose	2 (5.9)	5 (21.7)	6 (17.6)	0.156
Sore throat	10 (29.4)	3 (13.0)	8 (23.5)	0.354
Muscle soreness	1 (2.9)	3 (13.0)	4 (11.8)	0.364
Headache	4 (11.8)	3 (13.0)	1 (2.9)	0.364
Diarrhea	3 (8.8)	1 (4.3)	4 (11.8)	0.739
Conjunctivitis	0	1 (4.3)	0	–

BMI, body mass index; SBP, systolic blood pressure; DBP, diastolic blood pressure; COPD, chronic obstructive pulmonary disease.

* P < 0.05.

**Table 2:** Hospital course of COVID-19 patients with different vaccination does.

	**Unvaccinated** **(*n* = 34)**	**Partially vaccinated** **(*n* = 23)**	**Fully vaccinated** **(*n* = 34)**	***p*** **-value**	***p*****-value** **adjusted for** **age**	***p*****-value adjusted** **for age, sex,** **education**
Severity (mild/moderate/severe)	3/31/0	2/21/0	10/24/0	0.055	–	
Time to seroconversion (days)	27.3 ± 6.7	24.9 ± 5.6	24.7 ± 7.2	0.169	0.162	0.042^*^
**Laboratory findings**						
Viral titer (Ct value)						
N gene	20.6 ± 6.1	19.0 ± 5.9	22.5 ± 7.6	0.565	0.297	0.416
ORF1ab gene	23.7 ± 5.4	22.2 ± 6.5	25.6 ± 7.4	0.259	0.270	0.439
WBCs (×10^9^/L)	4.8 ± 1.5	5.1 ± 1.7	5.4 ± 2.0	0.407	0.557	0.980
Neutrophils (×10^9^/L)	5.2 ± 12.2	3.5 ± 1.4	5.2 ± 11.1	0.776	0.786	0.831
Lymphocytes (×10^9^/L)	1.2 ± 0.5	1.1 ± 0.5	2.8 ± 6.7	0.177	0.037^*^	0.048^*^
Monocytes (×10^9^/L)	0.4 ± 0.2	0.5 ± 0.2	1.1 ± 2.8	0.325	0.520	0.694
PLTs (×10^9^/L)	145.9 ± 49.3	142.5 ± 64.7	173.8 ± 69.2	0.093	0.208	0.845
Hemoglobin (g/L)	127.8 ± 13.6	136.9 ± 15.1	136.1 ± 17.6	0.045	0.124	0.891
FBG (mmol/L)	6.5 ± 3.1	5.6 ± 1.7	6.2 ± 3.3	0.555	0.549	0.409
TG (mmol/L)	1.5 ± 1.0	1.5 ± 0.9	1.3 ± 0.9	0.734	0.237	0.863
TC (mmol/L)	3.7 ± 1.0	4.2 ± 0.7	4.2 ± 1.0	0.147	0.837	0.681
ALT (U/L)	31.4 ± 25.6	27.4 ± 30.5	23.6 ± 11.6	0.379	0.301	0.341
TBIL (μmol/L)	12.3 ± 5.3	12.9 ± 5.3	10.6 ± 3.3	0.151	0.227	0.091
Interlukin-6 (pg/mL)	24.1 ± 26.8	21.0 ± 27.5	14.1 ± 18.8	0.297	0.588	0.772
**Imaging findings**						
Chest CT score	8.1 ± 5.2	5.7 ± 2.3	3.7 ± 3.7	<0.001^*^	<0.001^*^	0.001^*^

WBC, white blood cell; PLT, platelet; FBG, fasting blood glucose; TG, triglyceride; TC, total cholesterol; ALT, alanine transaminase; TBIL, total bilirubin.

* P < 0.05.

### Cognition, quality of life, and mental health evaluations

The results of the neuropsychological assessments are shown in Table 3. Cognitive impairment (MMSE < 25) was found in 15.4% of patients (26.5% of unvaccinated patients, 8.7% of partially vaccinated, and 5.6% of fully vaccinated patients). There was no significant difference in the MMSE score among the three groups with adjustment for age, sex, and education (*P* = 0.076). Quality of life was impaired in 30.7% of patients (38.2% of unvaccinated patients, 26.1% of partially vaccinated, and 26.5% of fully vaccinated). Among the three groups, unvaccinated patients had the lowest SF-36 total score (*P* = 0.042), especially in the MCS (*P* = 0.019) (Figures 1C,D). PTSD was diagnosed in 6.6% (6/91) of patients. Depression and anxiety were reported in 5.5% (5/91) and 6.6% (6/91) of patients, respectively. However, there was no significant difference in the incidence of PTSD, depression, or anxiety among the three groups.

**Table 3:** Cognition, quality of life and emotional state in COVID-19 patients with different vaccination statuses.

	**Unvaccinated** **(*n* = 34)**	**Partially vaccinated** **(*n* = 23)**	**Fully vaccinated** **(*n* = 34)**	***p*** **-value**	***p*****-value** **adjusted for** **age**	***p*****-value adjusted** **for age, sex,** **education**
**Cognitive function**						
SCD questionnaire score	3.8 ± 2.4	2.3 ± 1.9	3.5 ± 2.3	0.076	0.095	0.114
MMSE score <25	9 (26.5)	2 (8.7)	2 (5.9)	0.070	–	–
MMSE score	26.2 ± 3.6	28.7 ± 2.1	28.4 ± 2.3	<0.001^*^	0.013^*^	0.076
**Quality of life measures**						
SF-36 total scores	111.2 ± 16.1	122.2 ± 12.6	120.2 ± 11.5	0.018^*^	0.032^*^	0.042^*^
SF-36 impaired	13 (38.2)	6 (26.1)	9 (26.5)	0.508	–	–
Physical component summary	60.4 ± 6.9	63.7 ± 5.6	63.3 ± 5.7	0.139	0.290	0.338
Physical functioning	26.6 ± 3.6	27.7 ± 4.3	28.3 ± 1.9	0.099	0.437	0.613
Role-physical	6.0 ± 1.9	7.2 ± 1.4	6.7 ± 1.7	0.066	0.129	0.119
Bodily pain	9.7 ± 1.6	9.2 ± 1.3	9.9 ± 1.5	0.247	0.223	0.231
General health	18.1 ± 3.4	19.7 ± 2.8	18.4 ± 3.0	0.977	0.227	0.187
Mental component summary	50.8 ± 10.4	58.5 ± 8.3	56.9 ± 7.8	0.013^*^	0.015^*^	0.019^*^
Validity	15.9 ± 4.4	19.8 ± 3.3	19.4 ± 2.6	0.002^*^	0.001^*^	0.001^*^
Social functioning	7.9 ± 2.5	8.9 ± 2.0	8.8 ± 2.0	0.240	0.236	0.238
Role-emotional	5.0 ± 1.4	5.5 ± 1.0	5.3 ± 1.2	0.528	0.509	0.727
Mental health	22.0 ± 4.8	24.3 ± 4.3	23.4 ± 4.0	0.404	0.195	0.234
**Emotional state**						
PTSD, PCL-5 score	12.3 ± 16.7	9.0 ± 11.5	10.2 ± 12.6	0.911	0.762	0.964
PTSD, PCL-5 > 32	4 (11.8)	1 (4.3)	1 (2.9)	0.494	–	–
Depression, HADS-D	3.5 ± 3.5	4.7 ± 4.0	4.2 ± 3.5	0.088	0.533	0.341
Depression, HADS-D > 7	3 (8.8)	6 (26.1)	5 (14.7)	0.230	–	–
Depression, HADS-D > 10	1 (2.9)	2 (8.7)	2 (5.8)	0.844	–	–
Anxiety, HADS-A	4.7 ± 4.8	4.6 ± 3.6	4.4 ± 3.4	0.841	0.871	0.961
Anxiety, HADS-A > 7	4 (11.8)	4 (17.4)	6 (17.6)	0.812	–	–
Anxiety, HADS-A > 10	3 (8.8)	1(4.3)	2 (5.9)	0.881	–	–

Anxiety and depression (HADS-A, HADS-D) were considered as slightly increased when the score was >7 and increased when the score was >10. SCD, subjective cognitive decline; MMSE, Mini-Mental State Examination; SF-36, 36-item Short Form; PCL-5, Posttraumatic Stress Disorder Checklist−5; HADS-A, Hospital Anxiety and Depression Scale–Anxiety subscale; HADS-D, Hospital Anxiety and Depression Scale–Depression subscale.

* P < 0.05.

### Risk factors related to impaired quality of life

A total of 28 (30.7%) patients had impaired quality of life. Compared with fully vaccinated group, the unvaccinated group had a significantly higher risk of impaired quality of life (OR: 3.210, 95% CI: 1.008–10.220, *P* = 0.048). In addition, higher scores on the SCD questionnaire (OR: 1.430, 95% CI: 1.126–1.816, *P* = 0.003), HADS-D (OR: 1.229, 95% CI: 1.061–1.424, *P* = 0.006), HADS-A (OR: 1.219, 95% CI: 1.055–1.409, *P* = 0.007), and PCL-5 (OR: 1.062, 95% CI: 1.013–1.113, *P* = 0.013) were all associated with impaired quality of life. Higher chest CT scores were also related to impaired quality of life (OR: 1.181, 95% CI: 1.027–1.357, *P* = 0.02) (Table 4). In the multivariable model, the risk of impaired quality of life was more significant in the unvaccinated group than in the vaccinated groups (OR: 6.460, 95% CI: 1.414–29.517, *P* = 0.016). Higher scores on the SCD questionnaire (OR: 1.512, 95% CI: 1.124–2.034, *P* = 0.006) and HADS-D (OR: 1.294, 95% CI: 1.011–1.655, *P* = 0.04) were also associated with impaired quality of life. No significant effect of partial vaccination was found in either the univariable model or multivariable model (Table 4).

**Table 4:** Univariate and multivariate analyses of risk factors for impaired quality of life.

	**Univariate**		**Multivariate**	
	**OR** **(95% CI)**	***p*** **-value**	**OR** **(95% CI)**	***p*** **-value**
Age, >60 years	1.882 (0.677, 5.231)	0,225		
Sex, male	0.947 (0.363, 2.475)	0.912		
Education level (years)	0.977 (0.860, 1,110)	0.720		
BMI (kg/m^2^)	1.045 (0.888, 1.230)	0.595		
Current smoking	0.912 (0.200, 4.160)	0.905		
Hypertension	1.554 (0.581, 4.154)	0.380		
Diabetes	1.193 (0.365, 3.903)	0.770		
Dyslipidemia	0.705 (0.229, 2,164)	0.541		
Malignant tumor	5.040 (0.497, 51.116)	0.171		
**Vaccination**				
Partially vaccinated	0.952 (0.276, 3.286)	0.938	1.431 (0.314, 6.534)	0.643
Unvaccinated	3.210 (1.008, 10.220)	0.048^*^	6.460 (1.414, 29.517)	0.016^*^
SCD questionnaire score	1.430 (1.126, 1.816)	0.003^*^	1.512 (1.124, 2.034)	0.006^*^
MMSE score	1.001 (0.837, 1.196)	0.994		
PCL-5 score	1.062 (1.013, 1,113)	0.013^*^	1.011 (0.936, 1.092)	0.782
PTSD, PCL-5 > 32	3.417 (0.582, 20.07)	0.174		
Depression, HADS-D	1.229 (1.061, 1.424)	0.006^*^	1.294 (1.011, 1.655)	0.040^*^
Depression, HADS-D > 7	3.600 (1.058, 12.244)	0.040^*^		
Depression, HADS-D > 10	7.000 (0.739, 66.279)	0.090		
Anxiety, HADS-A	1.219 (1.055, 1.409)	0,007^*^	1.039 (0.814, 1.327)	0.758
Anxiety, HADS-A > 7	2.467 (0.750, 8.109)	0.137		
Anxiety, HADS-A > 10	3.417 (0.582, 20.070)	0.174		
Chest CT score	1.181 (1.027, 1.357)	0.020^*^	1.177 (0.976, 1.419)	0.088
WBCs (×10^9^/L)	1.113 (0.827, 1.498)	0.481		
Neutrophils (×10^9^/L)	0.961 (0.872, 1.058)	0.417		
Lymphocytes (×10^9^/L)	1.036 (0.932, 1.151)	0.516		
Monocytes (×10^9^/L)	2.200 (0.284, 17.058)	0.451		
PLTs (×10^9^/L)	1.001 (0.994, 1.009)	0.771		
Hemoglobin (g/L)	0.998 (0.968, 1.030)	0.911		
FBG (mmol/L)	0.872 (0.710, 1.070)	0.190		
TG (mmol/L)	1.010 (0.589, 1.730)	0.972		
TC (mmol/L)	0.987 (0.528, 1.842)	0.966		
ALT (U/L)	1.002 (0.983, 1.022)	0.830		
TBIL (μmol/L)	0.972 (0.877, 1.078)	0.591		
Interlukin-6 (pg/mL)	1.020 (0.988, 1.053)	0.215		

SCD, subjective cognitive decline; MMSE, Mini-Mental State Examination; PCL-5, Posttraumatic Stress Disorder Checklist−5; HADS-A, Hospital Anxiety and Depression Scale–Anxiety subscale; HADS-D, Hospital Anxiety and Depression Scale–Depression subscale; WBC, white blood cell; PLT, platelet; FBG, fasting blood glucose; TG, triglyceride; TC, total cholesterol; ALT, alanine transaminase; TBIL, total bilirubin.

* P < 0.05.

### Relationships of quality of life with the chest CT score and time to seroconversion

**Figure 2:**
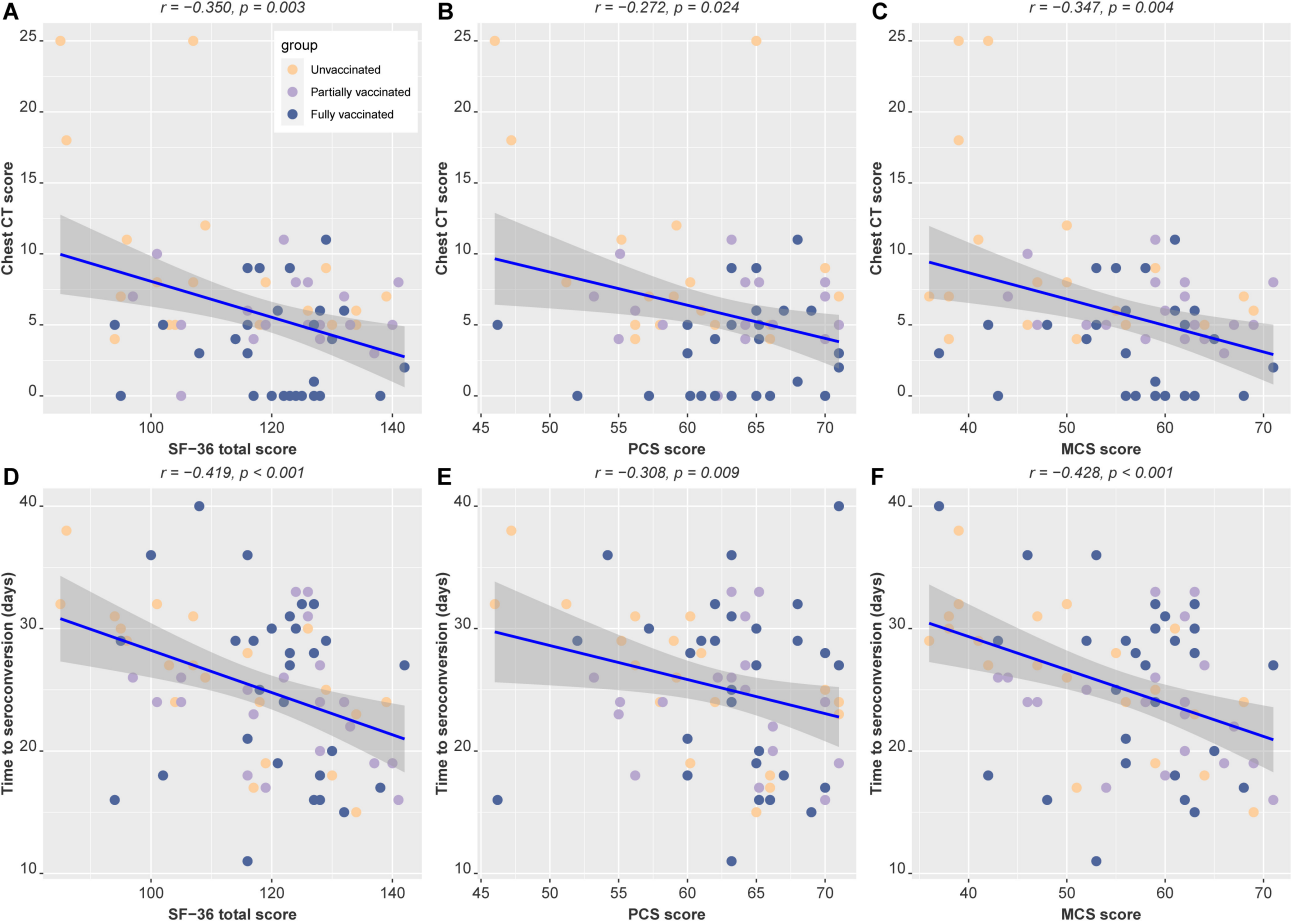
Partial correlation analyses of quality of life with the chest CT score and time to seroconversion, with age, sex and education as covariables. The color of point represents vaccination status. The regression line for all subjects (blue line) and upper and lower 95% CIs (gray shades) are shown. **(A–C)** Partial correlation analysis between chest CT scores and SF-36 total score, PCS score, and MCS score. **(D–F)** Partial correlation analysis between seroconversion time and SF-36 total score, PCS score, and MCS score. PCS, physical component summary; MCS, mental component summary.

Results of partial correlation analyses are shown in Figure 1. There were significantly negative associations of SF-36 scores with chest CT scores (*r* = −0.350, *P* = 0.003) and time to seroconversion (*r* = −0.419, *P* < 0.001) (Figures 2A,D). The same correlation was seen either in the PCS or MCS scores (Figures 2B,C,E,F). These results suggest that better quality of life was correlated with less severe lung lesions and shorter time to seroconversion during hospitalization.

### Mediation analysis

**Figure 3:**
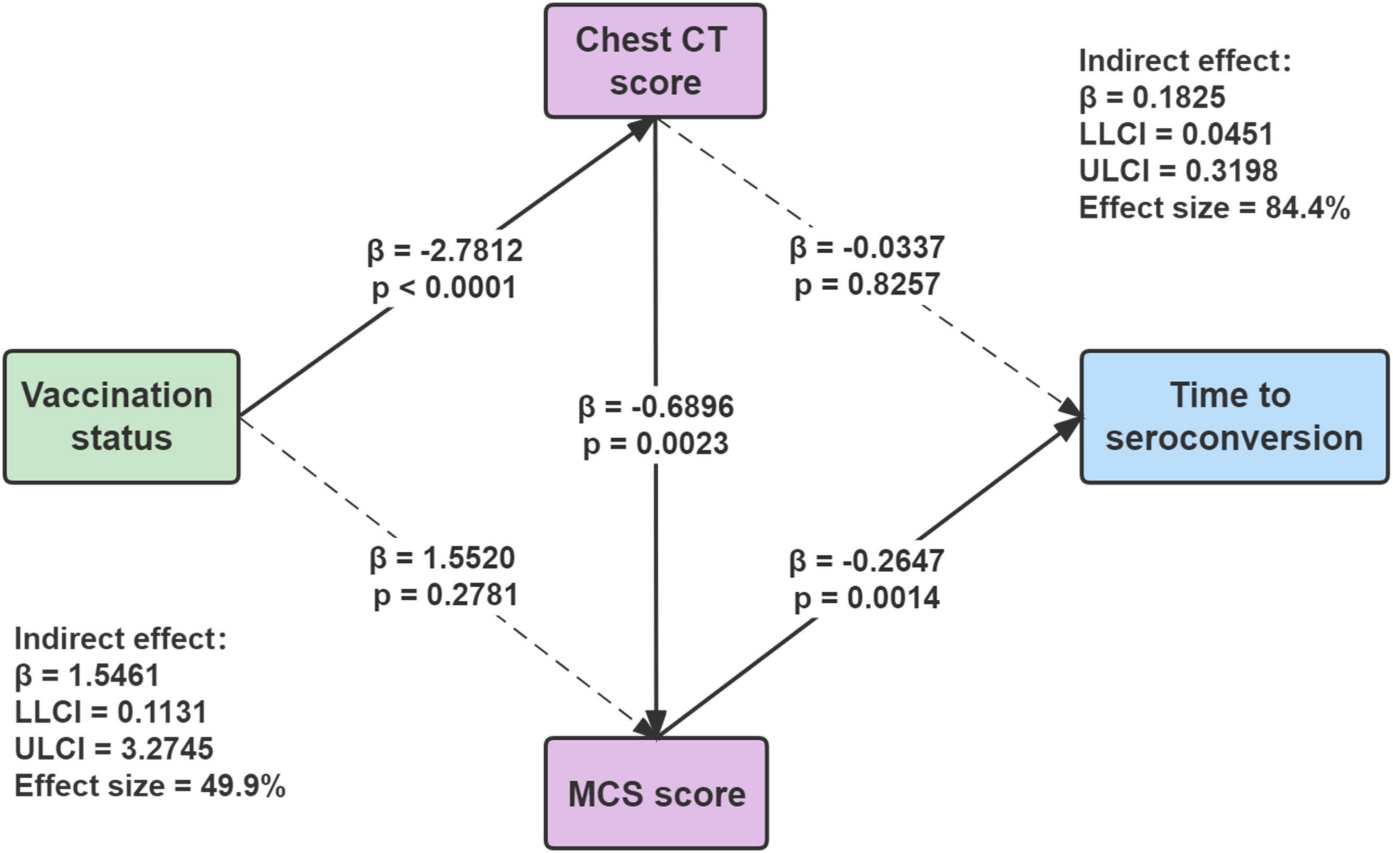
Mediation analyses for the association among vaccination status, chest CT score, MCS score, and time to seroconversion. Standardized β-coefficient was derived from mediation models controlling for age, sex, and education. Values are standardized path coefficients with 95% CIs in parentheses. The effect size of mediation was estimated from the proportion of indirect effect in the total effect. MCS, mental component summary. ULCI, upper limit of the 95% confidence interval; LLCI, lower limit of the 95% confidence interval.

Mediation analyses with correction for age, sex and education were performed to determine the associations of vaccination status with quality of life, chest CT score, and time to seroconversion. Figure 3 showed that chest CT score mediated the correlation between vaccination status and MCS score [indirect effect = 1.5461; 95% bootstrap CI (0.1131, 3.2745)]. Meanwhile, MCS score also mediated the association between chest CT score and time to seroconversion [indirect effect = 1.1825; 95% bootstrap CI (0.0451, 0.3198)] (Figure 3). In our current samples, there is no evidence of direct effect of vaccination status on the time to seroconversion, but vaccination could influence time to seroconversion by affecting CT score and MCS score indirectly.

## Discussion

The rapid spread and mutation of SARS-CoV-2 have attracted significant public attention, with concerns about increased transmissibility, increased severity of disease, and reduced diagnostic capacity. The mutation frequency and transmission speed of SARS-CoV-2 have rapidly increased. Breakthrough infections due to new variants after vaccination have been increasingly reported, even in mass-vaccination regions (25, 26), especially during the Delta variant epidemic. Therefore, there is a question about whether herd immunity bolstered by vaccination with an inactivated vaccine in China can protect against new variants and help promote rapid recovery with few COVID-19 complications.

Previous work on the long-term impact of COVID-19 on mental health is extensive (27–29) and has shown that functional mobility impairment, pulmonary abnormalities, and mental health disorders are the most common postacute sequelae of COVID-19 infection (30). However, these studies were usually meta-analyses and did not consider vaccination status. Additionally, short-term SARS-CoV-2 vaccine effectiveness in hospitalized patients has been rarely reported. In this study, we focused on vaccination effects in patients who had recently been admitted to the hospital. Our results indicated that fully immunized patients had significantly less severe chest CT findings, as represented by CT scores, than unvaccinated patients. It is possible that the vaccine protected against the progression of the disease from mild to severe (31).

After adjustment of age, sex and education, the three groups have no difference on the MMSE scores. However, the SF-36 scores and MCS scores of the three groups still had significant differences after correcting for age, sex, and education. The unvaccinated group had the lowest SF-36 total score and MCS score compared with the vaccinated group. Among patients recently infected with SARS-CoV-2 and who are under a closed management environment in the hospital, unvaccinated patients may suffer more mental stress, causing quality of life changes. Studies have revealed that vaccination-related stress levels significantly decrease after vaccination (32), and our data support this finding. The univariate and multivariate analyses revealed that vaccination status, cognitive impairment, depression, anxiety, and the chest CT score were significant risk factors for quality of life impairment in hospitalized patients.

The major outcome of this study was the relationship between vaccination status and seroconversion time. The negative associations between quality of life (SF-36 score) with the chest CT score indicated that better quality of life was correlated with less severe lung lesions. COVID-19 causes a wide impairment of diffusion capacity and restrictive ventilatory defects, which exist even at the time of hospital discharge (23, 33). Moreover, the negative correlations between the SF-36 score and time to seroconversion suggest that quality of life at baseline can significantly affect disease prognosis. Mediation analysis (24, 34) allowed us to observe the direct and indirect effects of each variable and explore whether vaccination status was independently associated with the effects of imaging and mental health. The mediation model suggested that vaccination status could influence time to seroconversion by affecting CT score and MCS score indirectly. It can be speculated that a higher quality of life at the outset leads to fewer lung lesions and shorter seroconversion time. Overall, full vaccination showed positive effects on viral clearance and the patient's recovery.

There are some limitations of our study. First, we confirmed that both the chest CT score and MCS score had mediating effects on the relationship between vaccination status and seroconversion time, but the observation windows were narrow because they were restricted to the hospitalization period. Follow-up data may be needed to confirm the persistence of the mediation effects in the long term. Second, only vaccination with inactivated vaccines were considered in this study, and whether this mediating effect exists for other kinds of vaccines still needs to be explored. Third, the sample size was relatively small due to the long period of neuropsychological assessments.

In conclusion, we found a full immunization course with an inactivated vaccine effectively decreases the chest CT score and improves the quality of life in hospitalized patients. Vaccination status could influence time to seroconversion by affecting CT score and MCS score indirectly. Our study highlights the importance of continuous efforts in encouraging a full vaccination course.

## Data availability statement

The raw data used and analyzed in the current study are available from the corresponding author on reasonable request.

## Ethics statement

The studies involving human participants were reviewed and approved by the Ethics Committee of the Second Hospital of Nanjing, Nanjing, China (Nr: 2021-LS-ky031). The patients/participants provided their written informed consent to participate in this study.

## Author contributions

WZ contributed to the study design and statistical analyses and wrote the manuscript. QC and JLu contributed to the revision of the manuscript. JD, JLi, and YY contributed to participants' recruitment and data collection. LF, XL, JLiu, JLiuf, and CL contributed to the data collection. BZ is the guarantor of this work and, as such, had full access to all the data in the study and takes responsibility for the integrity of the data and the accuracy of the data analysis, supervised the entire study, and reviewed the manuscript. All authors contributed to the article and approved the submitted version.

## Funding

This work was supported by the Jiangsu Funding Program for Excellent Postdoctoral Talent, the National Natural Science Foundation of China (81720108022), the Fundamental Research Funds for the Central Universities, Nanjing University (2020-021414380462), the Key Scientific Research Project of Jiangsu Health Committee (K2019025), Industry and Information Technology Department of Nanjing (SE179-2021), Educational Research Project of Nanjing Medical University (2019ZC036), and the Project of Nanjing Health Science and Technology Development (YKK19055). The funders had no role in the study design, data collection and analysis, decision to publish, or preparation of the manuscript.

## Conflict of interest

The authors declare that the research was conducted in the absence of any commercial or financial relationships that could be construed as a potential conflict of interest.

## Publisher's note

All claims expressed in this article are solely those of the authors and do not necessarily represent those of their affiliated organizations, or those of the publisher, the editors and the reviewers. Any product that may be evaluated in this article, or claim that may be made by its manufacturer, is not guaranteed or endorsed by the publisher.
